# RapidPlan knowledge based planning: iterative learning process and model ability to steer planning strategies

**DOI:** 10.1186/s13014-019-1403-0

**Published:** 2019-10-30

**Authors:** A. Fogliata, L. Cozzi, G. Reggiori, A. Stravato, F. Lobefalo, C. Franzese, D. Franceschini, S. Tomatis, M. Scorsetti

**Affiliations:** 1 Radiotherapy Department, Humanitas Research Hospital and Cancer Center, Via Manzoni 56, 20089 Rozzano, Milan, Italy; 2 Department of Biomedical Sciences, Humanitas University, Milan, Rozzano Italy

**Keywords:** Knowledge based planning, RapidPlan

## Abstract

**Purpose:**

To determine if the performance of a knowledge based RapidPlan (RP) planning model could be improved with an iterative learning process, i.e. if plans generated by an RP model could be used as new input to re-train the model and achieve better performance.

**Methods:**

Clinical VMAT plans from 83 patients presenting with head and neck cancer were selected to train an RP model, CL-1. With this model, new plans on the same patients were generated, and subsequently used as input to train a novel model, CL-2. Both models were validated on a cohort of 20 patients and dosimetric results compared. Another set of 83 plans was realised on the same patients with different planning criteria, by using a simple template with no attempt to manually improve the plan quality. Those plans were employed to train another model, TP-1. The differences between the plans generated by CL-1 and TP-1 for the validation cohort of patients were compared with respect to the differences between the original plans used to build the two models.

**Results:**

The CL-2 model presented an improvement relative to CL-1, with higher R^2^ values and better regression plots. The mean doses to parallel organs decreased with CL-2, while D_1%_ to serial organs increased (but not significantly). The different models CL-1 and TP-1 were able to yield plans according to each original strategy.

**Conclusion:**

A refined RP model allowed the generation of plans with improved quality, mostly for parallel organs at risk and, possibly, also the intrinsic model quality.

## Background

The clinical implementation of intensity modulated radiotherapy (IMRT) and volumetric modulated arc therapy (VMAT) techniques increased steadily in the last two (IMRT) or one (VMAT) decades [1]. Both IMRT and VMAT aim to deliver the optimal dose distribution computed by means of an inverse planning process. To achieve this, the optimization engines utilize numerical objectives derived from clinical aims (dose-volume relations linked to the management of complication or control probabilities). In practice, the nature of the inverse optimization, particularly for the presence of trade-offs between conflicting objectives, might require several iterations, and could be time consuming and highly dependent on operator’s skills [2, 3], inducing also high variability in the plan quality [4]. Some of the planning challenges relate to the difficulty in translating the clinical aims into effective optimization objectives. The case of IMRT (or VMAT) based treatment of locally advanced head and neck cancer is a paradigmatic example presenting many complexities: from the dose prescription to the presence of numerous critical structures very close to, or overlapping the target volumes.

To simplify and speed up the treatment planning process, while increasing the quality of the treatment plans, different research paths were investigated in the recent past, as the planning automation [5, 6], the knowledge based planning (KBP) [7, 8], or the multicriteria optimization [9, 10]. The KBP approach consists in the elaboration of DVH predictive models based on statistical analysis of historical data, i.e. treatment plans of good quality [7, 8]. A training process aims to build a mathematical model, which can be used to predict, for any new case (patient) with its own specificity, the optimal dose distribution. A comprehensive overview of the different methods for intensity modulated planning automation approaches, including KBP, has been published by Hussein et al. [11].

Focusing the attention on the KBP process, its efficacy relies on: i) the quality of the data used for the training, ii) the regression applied to build the predictive models, and iii) the consistency between the new case and the population used for the training (i.e. the generalization power of the model). The RapidPlan (RP) engine (Varian Medical Systems, Palo Alto, USA) is one commercial KBP tool, implemented in the Varian Eclipse treatment planning system. It has been widely studied in recent years, applied on different sites: liver [12], pelvis [2, 13], oesophagus [14], head and neck [15, 16], breast [17], lung SBRT [18], spine SBRT [19]. In summary, the evidence derived from published studies, demonstrated that the use of RP allowed a general improvement in the inter-patient consistency of the treatment plans, their intrinsic quality and the efficiency (time and workflow) of the process. It was proven that with the RP KBP approach, it was also possible to harmonize the practice among different centers (e.g. in a network) or among planners with different skills [2, 3]. KBP methods were explored also as a plan quality assurance tool, steering plans to better adhere to clinical trial criteria aiming to prevent poor clinical results [20–22].

RP is a machine learning process, and can assist the planner in achieving optimal dose distributions. Although the today’s achieved plan quality with the use of advanced technologies is of high level, any better understanding of the used technique that could lead to some improvement, is worth to be explored. The primary aim of the present work was to determine if the RP learning process could improve itself, i.e. if plans generated by an RP model trained on a set of good clinical plans, could be used as a new input set to re-train the model (in an iterative process), ending with a kind of optimized model with a manually driven feed-back learning process. The second aim of the study was to assess if models based on different input plans, optimized with different strategical criteria, are able to generate plans according to the specific original plan criteria. For this second point, models using the same patients were configured, and plans were optimized with different dose objectives reflecting the different criteria. The study case adopted was locally advanced head and neck cancer.

## Materials and methods

The RapidPlan KBP approach implemented in the Eclipse is briefly described in Appendix.

### RapidPlan models, CL-1 and CL-2

Eighty-three patients presenting advanced HNC, stage III-IV, treated from 2010 to 2014, were selected from the department database. Sixteen had nasopharyngeal, 41 oropharyngeal, 26 hypopharyngeal or laryngeal tumours. The choice of those 83 patients was based on their plan quality, that was considered, form the clinical viewpoint, as optimal for the institutional strategy, both for target coverage (as first priority) and critical structures sparing. A CT-scan was acquired for each patient in supine position (immobilized with a thermoplastic mask), with 3 mm adjacent slice spacing. Clinical target volumes (CTV) for elective and boost regions were delineated according to internationally accepted guidelines [23–25]. An isotropic 5 mm margin was added to CTV to obtain the planning target volumes (PTV). PTVs were finally cropped 4 mm inside the body contour. Organs at risk contouring was checked for all the patients for consistency, apart of the spinal cord length only, that was kept according to the clinical routine way to work. All plans for all patients were optimized for VMAT delivery (in the RapidArc form), with two to four full arcs (with individualized collimator angles, set according to the target and anatomical complexity of the cases). The plans were optimized for photon beams of 6 MV beam quality generated by Varian linacs (either Edge, TrueBeam, Clinac, Unique) as available in the department. For all the patients, the clinical plans selected for the model training were optimized by means of the Progressive Resolution Optimizer (PRO) engine while the final calculations were made by means of the Anisotropic Analytical Algorithm AAA (PRO and AAA versions from 8.9 to 11). The dose was prescribed with a simultaneous integrated boost, with 54.45 and 69.96 Gy to the elective and the boost volumes, respectively, in 33 fractions. All the plans were normalized to the mean dose to the boost planning target volume PTV_boost. The treatment plans of those 83 patients were used to build an RP model. Patient and their anatomical characteristics are reported in Table 1.

**Table 1:** Patients’ characteristics

		Model
Number of patients	Patients	83
Tumour site	Nasopharyngeal	16 patients
	Oropharyngeal	41 patients
	Hypopharyngeal and laryngeal	26 patients
PTV_69.96Gy volume	Mean ± SD [range], cm^3^	270.3 ± 15.0 [31.3, 641.9]
PTV_54.45Gy volume	Mean ± SD [range], cm^3^	421.4 ± 18.4 [187.0, 1048.9]
Parotids volume	Mean ± SD [range], cm^3^	27.7 ± 0.7 [11.8, 72.9]
Oral cavity volume	Mean ± SD [range], cm^3^	126.1 ± 2.6 [37.0, 162.4]
Larynx volume	Mean ± SD [range], cm^3^	50.2 ± 3.1 [15.8, 99.5]
Thyroid volume	Mean ± SD [range], cm^3^	19.1 ± 1.5 [5.3, 59.9]
Spinal cord volume Spinal cord length	Mean ± SD [range], cm^3^ Mean ± SD [range], cm	33.3 ± 1.1 [12.3–61.4] 20.5 ± 0.6 [12.9–37.2]
Brain stem volume	Mean ± SD [range], cm^3^	21.6 ± 0.8 [9.4–37.0]

*SD* error of the mean

The primary aim of this work was to determine if an iterative learning process (in two steps) could improve the RP performances; the following procedure was adopted. The selected clinical plans were added to generate and train a baseline RP model, named CL-1, as described in Fogliata et al. [16]. In brief: the OARs included in the model were spinal cord, brain stem, oral cavity, parotids, submandibular glands, larynx, thyroid, eyes, mandible and constrictor muscles. Regarding the targets, PTV_boost was the PTV receiving 69.96 Gy, PTV_all was the whole PTV (whichever dose level), PTV_elective was the PTV_all deducted the PTV_boost with 4 mm margin in the axial directions only (the plane of gantry rotation). The optimization objectives used in the model, and already published in [16], are summarized in Table 2. Additionally, a manual normal tissue objective (NTO) was also included in order to shape the dose fall off outside the targets using the following parameters: distance from the target border 7 mm, fall off start/end doses 105%/5%, fall-off 0.80, priority 120. The choice of the 5% as end dose as NTO parameter allowed to reduce the neck dose below the 50% of the prescription dose with no additional structure delineation.

**Table 2:** Optimization objectives in the RapidPlan models (according to [16]). The term ‘generated’ is not fixed a priori, while it is determined by the model and estimated DVH

Structure	Objective	Volume [%]	Dose	Priority
PTV_all	Lower	100	99	110
PTV_boost	Upper	0	101	120
	Upper	0	100	120
	Lower	100	100	120
	Lower	100	99	120
PTV_elective	Upper	0	101	110
	Upper	0	100	110
	Lower	100	100	110
	Lower	100	99	110
Brain stem	Upper	0	Generated	90
	Line	Generated	Generated	Generated
Constrictors	Line	Generated	Generated	Generated
Eyes	Mean	–	Generated	Generated
	Line	Generated	Generated	Generated
Larynx	Mean	–	Generated	60
	Line	Generated	Generated	Generated
Mandible	Upper	5	Generated	Generated
	Line	Generated	Generated	Generated
Oral cavity	Mean	–	Generated	60
	Line	Generated	Generated	Generated
Parotids	Mean	–	Generated	70
	Line	Generated	Generated	Generated
Spinal cord	Upper	0	Generated	90
	Line	Generated	Generated	Generated
Submandibulars	Line	Generated	Generated	Generated
Thyroid	Line	Generated	Generated	Generated

In the second step of the work, new 83 plans for the same patients were obtained using the model CL-1. From an operational point of view, the plans in this set resulted from a double optimization run (the second round was applied to the dose distribution from the first round and was run from multiple resolution level 2) with no human interaction. The optimization and the dose calculation were performed by means of the Photon Optimizer and the Acuros-XB engines in the Eclipse 13.6 environment. The 83 plans newly generated were used to train a second model, named CL-2, as summarized in Table 3. This second model was built with the same optimization objectives as CL-1.

**Table 3:** Brief description of the plans used to train the four RapidPlan models. All are based on the same 83 head and neck patients, using the same geometry as the clinical plans

Model	Input plans for model training
CL-1	Clinical plans, manually optimized to achieve the goals
CL-2	Plans generated with RapidPlan model CL-1
TP-1	Plans generated with a simple template, no personalized optimization
TP-2	Plans generated with RapidPlan model TP-1

For all the models configured in this work, the following steps were assessed before proceeding to the model analysis. The structures or plans identified by the system at the end of the model training as influential points, or possible outliers (data that differs considerably, dosimetrically or geometrically, from the whole training set) were checked and evaluated case by case, as described in [16], to eventually exclude possible real outliers. No structures were excluded from any of the model. Then, a short verification of the DVH estimation relative to the input data was performed, both internally in the configuration program and with plans generated using the model for the patients used for training (often called closed-loop verification). Of those steps, no detailed results were here given, being a routine process of model configuration, out of the scope of the current work.

The quality of the two models (model quality) was analysed firstly by comparing the goodness-of-fit and goodness-of-model statistical parameters obtained at the end of the training phases. The relative performance quality of the two models, CL-1 and CL-2, i.e. their ability to produce high quality plans (clinical outcome quality), was investigated by means of a validation process. This consisted in a dose-plan comparison of the plans, optimized with each model, for the 20 validation patients chosen from the clinical database, presenting tumour and anatomical characteristics consistent with the cases used to train the models. The comparison was based on averaged dose parameters among all the validation patients. The Shapiro-Wilk test was used to test the normality of the data, and the statistical significance was evaluated with the two tailed paired Student t-test (the level of significance was set to 0.05).

The CL-1 model has been extensively validated, and detailed results on the validation phase have already been published [16] and not repeated in this report: the comparison between the original clinical plans and the RP plans showed a significant plan quality improvement with RP with reductions of 2, 5 and 10 Gy of the mean doses to the parotids, oral cavity and larynx, with a global normal tissue complication probability reduction of about 7%.

### RapidPlan models, TP-1 and TP-2

A third step in the study was the assessment of the relevance (if any) of the planning strategy adopted in the generation of the initial training set. To exploit this topic, for each of the original 83 patients, a third set of baseline plans was generated using the following simple static (not patient-tailored) dose-volume constraints template (defined as a list of predefined optimization objectives): parotids mean dose < 25 Gy with priority 70, oral cavity mean dose < 35 Gy priority 70 (only for non-oral cavity tumours), thyroid mean dose < 40 Gy priority 60, spinal cord max dose < 35 Gy priority 90, brain stem max dose < 54 Gy priority 80, targets minimum and maximum doses equal to the specific prescriptions with priority 100, automatic NTO priority 120. No attempt to improve the plan quality (no human interaction) was applied. In principle, the use of a fixed template for all cases might result in some sub-optimality of the plans but, in general, the plan quality achieved was considered clinically acceptable for all 83 cases. The PO and Acuros-XB (versions 13.6) engines were used for this phase.

This third cohort of 83 plans was used to configure and train a new RP model, named TP-1 (see Table 3). The differences of the 83 manually adjusted clinical plans used to configure the model CL-1, and this set of manual template-based plans for TP-1 were evaluated and reported to describe the relative figure of merit of the two planning strategies (the clinical one, and that according to the template).

To compare the clinical performances of the two models, CL-1 and TP-1, a validation experiment was carried out on the same 20 validation cases.

Finally, similarly to CL-2, a fourth model, TP-2, was built based on the plans generated with the TP-1 model (Table 3). Both TP-1 and TP-2 had the same set of optimization objectives as the previous CL models. Then, TP-1 and TP-2 models were compared, similarly to CL-1 and CL-2, on the same cohort of 20 validation patients.

## Results

### CL-1 to CL-2: the intrinsic model quality

The parameters summarizing the intrinsic model quality in mathematical terms are reported by the Model Configuration engine at the end of the model training. To present the goodness of the models, the R^2^ and MSE parameters described in the Appendix have been reported in Table 4. The same parameters of TP-1 and TP-2 are also included for comparison and completeness.

**Table 4:** Goodness of models parameters of all the four models, CL-1 and CL-2, TP-1 and TP-2. Description and interpretation of the parameters is given in Appendix

	Goodness-of-fit: R^2^				Goodness-of-estimation: MSE			
	CL-1	CL-2	TP-1	TP-2	CL-1	CL-2	TP-1	TP-2
Brain Stem	0.606	0.901	0.893	0.839	0.0211	0.0040	0.0045	0.0039
Constrictors	0.659	0.793	0.666	0.727	0.0256	0.0262	0.0189	0.0206
Eyes	0.743	0.781	0.866	0.805	0.0073	0.0010	0.0054	0.0087
Larynx	0.534	0.756	0.619	0.684	0.0131	0.0044	0.0069	0.0051
Mandible	0.747	0.836	0.763	0.835	0.0050	0.0029	0.0047	0.0034
Oral Cavity	0.599	0.866	0.474	0.818	0.0047	0.0028	0.0031	0.0030
Parotids	0.541	0.634	0.437	0.602	0.0037	0.0053	0.0029	0.0027
Spinal Cord	0.204	0.731	0.475	0.398	0.0256	0.0074	0.0029	0.0053
Submandibulars	0.582	0.744	0.596	0.721	0.0282	0.0289	0.0301	0.0271
Thyroid	0.706	0.892	0.722	0.809	0.0079	0.0043	0.0043	0.0055

The data reported in Table 4 showed on average an improvement for the CL-2 model with respect to CL-1 for what concerns the goodness-of-fit, R^2^; this is also reflected in a general improvement of the goodness-of-estimation parameter MSE, although not with the same intensity.

**Fig. 1:**
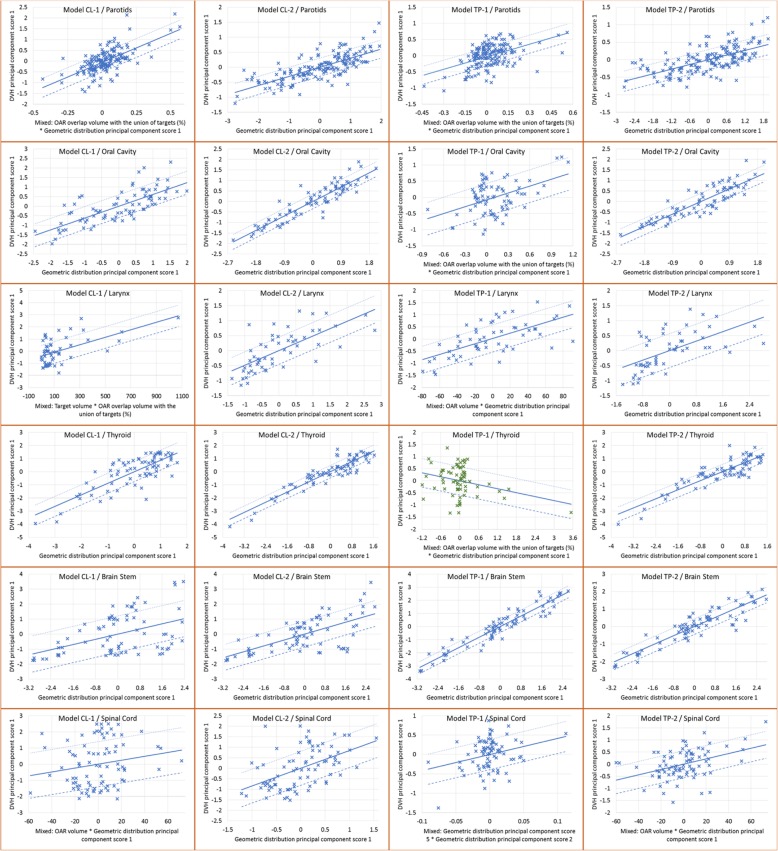
Regression plots for different OARs in the 4 models. In the rows, different OARs are represented, while in the column they refer to the different models: CL-1, CL-2, TP-1, TP-2, for a visual comparison

A deeper, although qualitative, evaluation of the regression plots is presented in Fig. 1 for the most salient OARs, where the data and regression fit of the geometric (abscissa in the plots) and dosimetric (ordinate in the plots) features of all the four models are presented for some of the OARs trained in the models. Data were exported through the Model Analytics cloud-based tool for DVH estimation model analysis.

Comparing CL-1 and CL-2 plots (first two columns in the figure), the difference in the geometric features (abscissa) used for the regression phase is noticeable, being the same (principal component score PCS-1) in all the CL-2 cases, while for larynx, parotids and spinal cord in the CL-1 model, it was a mix of two different geometrical features. In particular, the CL-1 larynx regression plot presented the points (the features from the input plans) sparsely spread in the ordinate axis (dosimetric feature) and clustered at low abscissa values, with the combined geometrical feature of target volume and OAR overlap volume to target. This clustering effect is not anymore present in CL-2, where the geometric distribution PCS1 is instead used as single geometric feature. In the model analysis from CL-1 to CL-2, the larynx showed an improved R^2^, and in particular a rather poor MSE (> 0.01) for CL-1, dropped down to < 0.01 with CL-2. The MSE reduction should firstly translate into a narrower DVH estimation bound using CL-2.

The spinal cord can be considered in a different way: CL-1 presented a mixed geometric feature, where also the OAR volume was included. The contouring definition of this structure is quite difficult for what concerns its length: it is not fully delineated, and its contour could extend few centimetres below the target delineation, or caudally toward the abdominal region, and its delineation in terms of length was not made homogeneous in this study, to reflect the clinical practice. It is a serial organ and the major concern is the maximum dose, while the entire volume is of very limited clinical interest. The inclusion of the OAR volume as geometric feature for spinal cord could mislead the clinical approach. To consider is that the PCA is not used to analyse the regions of the OAR out of the radiation fields (where a simpler approach is used instead); in the particular case of the spinal cord, were a non negligible amount of the structure is not seen by the fields, the use of the OAR volume as geometrical feature in the model might be considered as suboptimal for the clinical outcome. Anyway, in CL-2, the OAR volume was not anymore part of the geometric feature, with an improvement in the regression plot (visual), R^2^, and especially MSE, suggesting a better management of the geometrical feature in this refined model.

Similar evaluations are valid for the TP models. Of notice, the TP-1 model presented for the thyroid structure a decreasing regression line for a mixed geometric feature, with clustered data (although the R^2^ presented a value of 0.722). Moving from TP-1 to TP-2 the model improved: no more descending regression, non-clustered distribution, and increased R^2^ values.

### CL-1 to CL-2: the dosimetric validation of the models’ performance

**Fig. 2:**
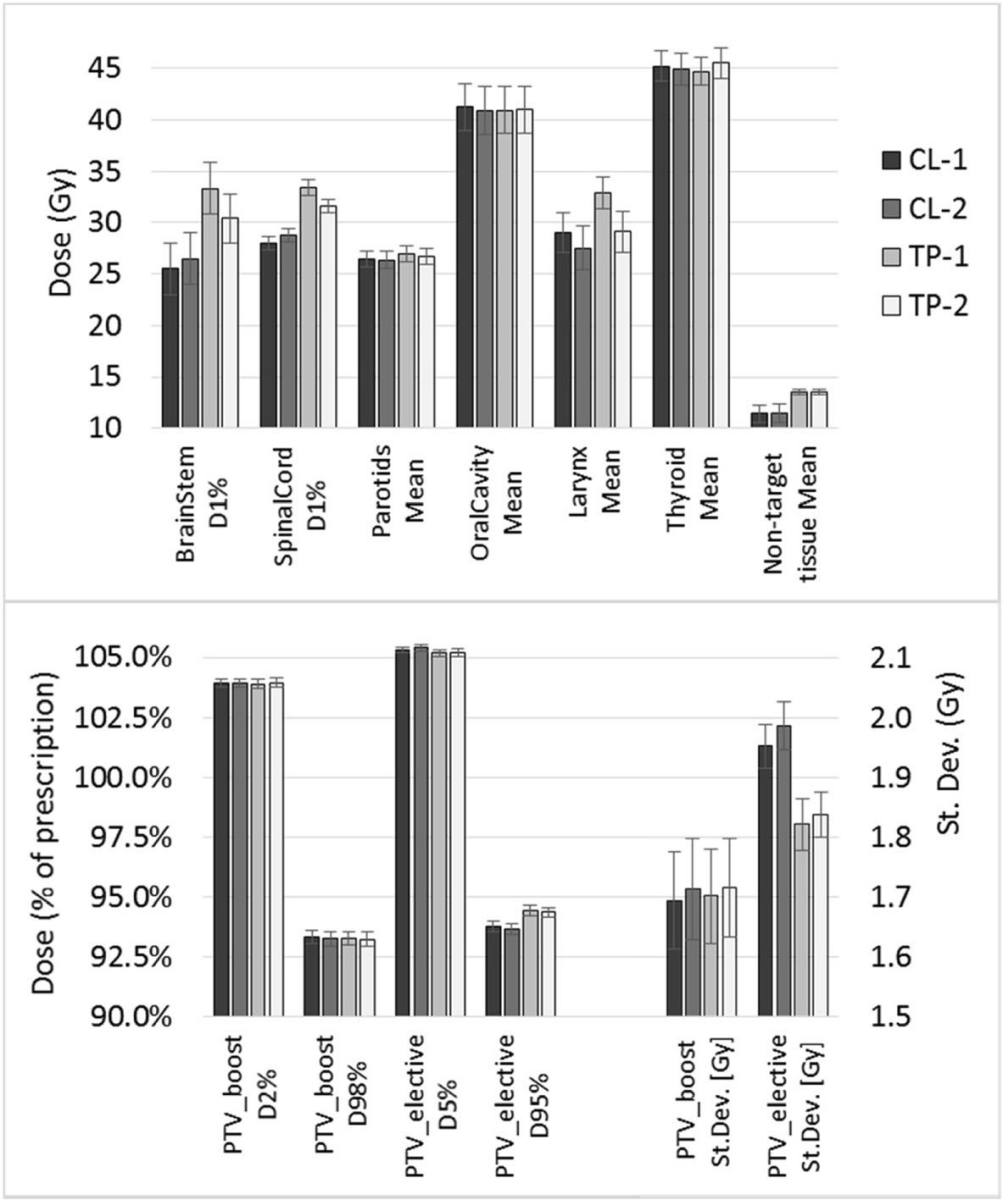
Dosimetric results, averaged on the 20 validation cases, for the four studied RapidPlan models (CL-1 and CL-2, TP-1 and TP-2). (a) Near-to-maximum (D_1%_) or mean doses to the most salient OARs and non-target tissue. (b) D_2%_ and D_98%_ for PTV_boost, D_5%_ and D_95%_ for PTV_elective, and standard deviation of the dose in the two targets, to evaluate the targets dose homogeneity

The dosimetric evaluation of the results is here reported for the OARs considered most salient in our clinical strategy, for the non-target tissue (patient contour from the whole CT dataset excluding all targets) and for the targets. In Table 5, the dose differences between various RP models for some parameters and structures were presented averaged over the 20 validation cases, together with their statistical significance. The specific dose values are reported for all the RP models in Fig. 2a for the OARs, for which the DVH estimation is part of the optimization process, and in Fig. 2b for the targets and the non-target tissue, where only the optimization objectives and the NTO are active in the plan optimization phase.

**Table 5:** Dose differences (averaged over the 20 validation patients) for some structures and target parameters, using different RapidPlan models. Error is reported as standard error of the mean. In parenthesis the *p* values. Targets and non-targe tissue have no DVH estimation from the RP process

	CL-2 – CL-1	TP-2 – TP-1	CL-1 – TP-1
BrainStem, D_1%_ (Gy)	3.8% ± 1.8% (0.054)	−8.8% ± 1.7% (< 0.001)	−29.6% ± 4.7% (< 0.001)
SpinalCord, D_1%_ (Gy)	2.7% ± 1.6% (0.119)	−5.5% ± 1.2% (< 0.001)	−19.0% ± 2.2% (< 0.001)
Parotids, Mean (Gy)	−0.5% ± 0.4% (0.159)	−0.9% ± 0.4% (0.029)	−1.8% ± 0.4% (< 0.001)
OralCavity, Mean (Gy)	−1.0% ± 0.3% (0.007)	0.1% ± 0.3% (0.713)	0.8% ± 0.2% (0.008)
Larynx, Mean (Gy)	− 5.1% ± 0.8 (< 0.001)	−11.5% ± 1.6% (< 0.001)	− 14.1% ± 1.8 (< 0.001)
Thyroid, Mean (Gy)	−0.5% ± 0.4% (0.277)	1.9% ± 0.4% (< 0.001)	1.1% ± 0.5% (0.042)
PTV_boost, D2% (Gy)	0.0% ± 0.1% (0.881)	0.0% ± 0.1% (0.209)	0.0% ± 0.1% (0.431)
PTV_boost, D98% (Gy)	−0.1% ± 0.1% (0.130)	−0.1% ± 0.1% (0.244)	0.0% ± 0.1% (0.551)
PTV_boost, St.Dev. (Gy)	1.1 ± 0.8% (0.208)	0.8% ± 0.7% (0.269)	−0.4% ± 1.0% (0.661)
PTV_elective, D5% (Gy)	0.1% ± 0.1% (0.093)	0.0% ± 0.1% (0.396)	0.1% ± 0.1% (0.077)
PTV_elective, D95% (Gy)	−0.1% ± 0.1% (0.092)	−0.1% ± 0.1% (0.307)	−0.7% ± 0.1% (< 0.001)
PTV_elective, St.Dev. (Gy)	1.8% ± 0.7% (0.021)	0.9% ± 0.7% (0.198)	6.6% ± 0.7% (< 0.001)
Non-target tissue, Mean (Gy)	0.4% ± 0.2% (0.094)	−0.1% ± 0.1% (0.647)	−18.6% ± 6.9% (0.017)

In a more clinical perspective of the validation phase, the analysis of the plans generated by CL-1 and CL-2 did not present a considerable improvement in their quality. In particular, for the serial organs, brain stem and spinal cord, an average increase of the D_1%_ of 3–4% was shown, although not significant. For those structures, the strongest optimization objective in the model was an upper objective with fixed high priority. This upper objective tends to force the reduction of the volume receiving a single dose value, not acting on the entire DVH dose range where the DVH estimation (and subsequent line optimization objective) is working. This could be a possible reason of missing improvement, if not deterioration, although the model parameters R^2^ and MSE improved, as is the case of the brain stem. On the contrary, the parallel organs presented lower mean doses of about 1%, with the larynx showing a mean dose reduction using CL-2 of about 5% in average (highly significant). This dosimetric effect on mean OAR dose could be seen in relation with the model improvement as MSE parameter for the specific structure. A higher MSE implies a better DVH estimation with the model; an improved DVH estimation would generate more achievable optimization objects; since the optimization objects are located below the DVH estimation bound, the clinical result of the model (the plan quality) might be improved relative to the estimation (as the case of the larynx).

### CL and TP models

The quality of the two plan cohorts used to build the CL-1 and TP-1 models are summarized in Table 6, as dosimetric parameters of the most relevant structures. A higher quality of the plans was assessed according to the trade-off strategies adopted in our institution in the CL-1 cohort, while the plans denoted for the TP-1 model were not optimized following any balance of OAR irradiation since the optimizer runs a simple static template with no human direction. The results of the single OARs cannot give a complete overview of the plan, whose quality depends also on the dose to any tissue surrounding the targets. For example, the non-target tissue volume receiving more than 40 Gy, reported in Table 6, is more than 50% higher for the TP plans, confirming the increased attention in the manual clinical plan optimization. However, the template gave to the oral cavity and the thyroid mean doses lower than the mean doses achieved by the CL plans, while all the other structures received higher doses (mimicking a different strategy).

**Table 6:** Dosimetric comparison between plans used to train CL-1 (clinical plans) and TP-1 (from simple template) models. Errors refer to the standard deviation of the mean. p is the significance value according to the Student t-test

Organ	Parameter	Plans for CL-1	Plans for TP-1	*p*	Difference TP-CL (%)
Brain Stem	D_1%_ (Gy)	29.1 ± 1.7	46.2 ± 1.4	< 0.001	+ 58.8
Spinal Cord	D_1%_ (Gy)	31.7 ± 0.8	36.5 ± 0.9	0.008	+ 15.1
Parotids	Mean (Gy)	25.9 ± 0.7	27.9 ± 0.5	< 0.001	+ 7.7
Oral Cavity	Mean (Gy)	44.1 ± 1.0	40.7 ± 0.8	< 0.001	−7.7
Larynx	Mean (Gy)	38.5 ± 1.7	50.2 ± 1.5	< 0.001	+ 30.4
Thyroid	Mean (Gy)	46.5 ± 1.1	44.5 ± 0.9	0.001	− 4.3
Non-target tissue	Mean (Gy)	7.6 ± 0.4	9.1 ± 0.4	< 0.001	+ 19.7
	V_40Gy_ (cm^3^)	666 ± 23	1031 ± 28	< 0.001	+ 54.8
PTV_boost	D_5%_ (%)	102.6 ± 0.6	103.0 ± 1.2	0.001	
	D_95%_ (%)	95.6 ± 1.0	94.9 ± 2.4	0.009	
PTV_elective	D_5%_ (%)	104.7 ± 1.2	104.4 ± 1.3	0.100	
	D_95%_ (%)	95.8 ± 1.1	96.9 ± 1.5	< 0.001	

To evaluate if the differences reported in Table 6 affected the RP model training and were reflected into the optimization of new cases, the comparison of the validation phases between CL-1 and TP-1 was performed and reported in the last column of Table 5. Both oral cavity and thyroid received (slightly) lower doses using the TP-1 model relative to the CL-1 model, according to the input plans. The largest differences in the plans obtained with the CL-1 model relative to the TP-1 model were the near-to-maximum dose to the brain stem and spinal cord, and the mean dose to the larynx, the organs that had a higher sparing in the initial plans feeding the CL-1 model. In summary, the model was able to yield plans according to the dosimetric strategy of the original input plans. A subsequent generation of a new model based on RP plans (CL-2 or TP-2) could, on the other hand, improve the specific model.

## Discussion

Different knowledge based predictive RP models were generated and analysed in the current work, in the frame of advanced head and neck cancer VMAT treatment. The first objective of this work aimed to determine if the performance of an RP model could be improved by re-training it with a set of plans originated by itself in an iterative learning process.

Many publications on RP models showed that, in various anatomical sites, the quality of KBP plans, on average, outperformed that of the corresponding clinically accepted plans [2, 12–18, 20]. The improvement observed in all the studies was, in part, due to the use of the line optimization objectives defined slightly below the estimated DVH lower bound, i.e. attempting to drive the optimization towards the best estimated DVH. Given this fact, the plans generated by RP could systematically result in better plan quality than the corresponding clinical plans used to train the model.

The experiments summarized in the present report showed that a general improvement in the intrinsic model quality, from the mathematical point of view, was obtained with CL-2 presenting overall better R^2^ and MSE values, and improved regression plots when compared to CL-1. This step of the model quality evaluation is a preamble needed to assess the model performance as quality of the plans generated by the models. In particular, attention could be paid in the interpretation of the results related to the goodness of model parameters. For example, the possible risk of overfitting should be considered: an R^2^ value too close to unity could be a symptom of overfitting. In this case, the model would present optimal goodness-of-fit parameters, but may not necessarily be able to generate good clinical treatment plans for patients other than the cohort used to train the model. Further models, say CL-n, might potentially induce a sort of overfitting, in the sense that every further model would lead to plans more and more similar to themselves, reducing the cohort plan variability. This risk could be avoided by using a large number of initial plans on patients with a wide spectrum of anatomical differences. This choice would also account for interorgan dependency, whose effect could change the model prediction performance, since machine learning algorithms approaches are data-driven, and data provenance and choice are hence of primarily importance [27].

The features identified in a model should capture the interpatient OAR variability, both in terms of anatomy and dose distribution. The anatomical interorgan variability is a matter of fact, and for this reason it is fundamental to cover the largest possible knowledge on a wide anatomical spectrum. On the contrary, the dosimetric variability is a consequence of the planning strategy: different plans from the clinical practice could follow different trade-offs between dose sparing goals of different OARs. Such a dosimetric variability cannot be simply expressed in the model. When the plans in the training process present the same strategy/trade-offs, the geometric and dosimetric features from the GED and DVH should be sufficient to predict consistent DVH, since the model input data well describe the goal in many situations. When there is a variation in the trade-offs (for example from interplanner or interorgan variability), the data from the DVH and GED could be not anymore sufficient to properly predict the DVH, and mixed geometric features are then used. In this sense, mixed geometric features in a model could be the symptom of a set of plans not fully binded in a common planning strategy (or structure delineation strategy, as it was the case of the spinal cord). A second iterative model, as CL-2 or TP-2, having input plans generated according to a strategy translated and summarized in the first model (CL-1 or TP-1), will possibly be able to not use patient specific anatomical features to predict the new DVH. In this sense, the iterative model could reduce the interpatient/interplan variability of the initial dataset. However, attention has to be paid also to a good description of the organ specific contouring. A comprehensive study on interpatient variation on OAR dose sparing is well described by Yuan et al. [28] from the Duke University group, whose studies are among the founding works of the DVH estimation as implemented in RP.

More relevant from the clinical viewpoint is the quality of the RP plans (model performance): the plans generated with the CL-2 model were superior to those from the CL-1 model in terms of the mean doses to the OARs, while for the near-to-maximum doses to the serial organs the data showed a small detriment. However, although in many cases significant, the specific differences were modest in absolute terms and should be correlated to limited clinical risks.

A plan quality improvement was also observed in some structures when TP-1 and TP-2 models were compared in the validation tests. The degree of plan improvement between those two models, the first generated with a simple template based plans and the second with RP plans as input, might depend on the distance of the input plans from an ideal Pareto surface. In the case of a model training performed with a set of input plans truly proximal to the Pareto surface, only small improvements could be possible, at a price of other organs’ irradiation. Indeed, this was confirmed (inversely) by the analysis of the TP based plans, which were not optimized case by case. The dosimetric results in the validation showed a larger variation between TP-1 and TP-2 for the structures that were less demanding in the objective template. This concept is similar to what published by Cagni et al. [29], where the authors assessed the RP accuracy of predicting DVH by training models with automatically generated Pareto optimal plans, in the attempt of minimizing the quality inconsistency of manually generated plans, or, as in the example of the current work, of plans generated without patient specific care, like the TP based plans.

Summarizing the first aim of this work, a second, iterative model could improve from the mathematical point of view, leading to a model potentially better predicting the DVH, although of no clinical clear impact. It might be worth to generate a second iterative model in case the first model presents mixed geometrical features.

The second objective of the present work aimed to clarify the planning strategy impact, by comparing a careful “manual” optimization with lots of human interaction and a rigid template-based optimization with no interactions to improve the plan quality: the RP model translated the dose distribution of the input plans into the quality of the new plans optimized prospectively through the model. The two different strategies of the manual plans CL and TP, summarised in Table 6, were reproduced by the respective RP models, for example in the brain stem, spinal cord and larynx doses. This result is a confirmation of the knowledge base planning concept, where the institutional plan quality personalized on the past patients is transferred to the new patients. With the KBP, this is actually accomplished not only across a list of desired dose achievements, but tailoring the dose distribution strategy to the new anatomy of the new patient.

All the mentioned points suggest that the plan and patient selection for any new RP model is crucial, since the RP based results will reproduce the quality and dosimetric strategy of the input plans, for the chosen patient anatomy ranges, as also suggested by Cagni et al. [29].

A potential critical point in an automated process, is the use of the same (or not) optimization and calculation algorithms for generating the plans used to feed the model, and the validation phase. In the present work, the clinical plans (for CL-1 models) were generated with the PRO optimization engine and the AAA algorithm, while in the whole RP validation and subsequent model, the PO optimization and Acuros dose calculation algorithms were used. It is known that PO was found to prevail over PRO for VMAT planning [30, 31], and Acuros is more accurate than AAA [32, 33]. Concerning the optimizer, outperforming PO with respect to PRO, could have a double effect. On one side, the initial clinical plans (used to generate the model CL-1) might have been better if optimized with PO; on the other side the improved quality of the RP generated plan could in part be ascribed to the different optimizer. This different characteristic of the two optimizer could be considered a limitation in the clinical practice. However, the algorithms differences should in principle have no real impact in the use of RP, whose core is the DVH estimation. This is especially for what concerns the optimization engine, up to the limit proposed by Cagni et al. [34], where the authors used dose distributions from Tomotherapy plans to generate an RP model, which was then used for VMAT planning with good results and comparisons. More delicate is the dose calculation algorithms, where, for the same plan, different dose distributions could be computed. However, in this work, all the comparisons were between plans consistently generated by the PO optimizer, and computed with Acuros. The possible critical point is the first model (CL-1), where the clinical plans were computed with AAA. However, this reflects the real clinical work, and we decided to keep the original plans for this study, according to the dose distribution that was clinically accepted for patient treatment, aware that those plans could have been slightly different with a different dose calculation algorithm. This small effect was not affecting the two TP models, where the same combination of PO and Acuros was always used.

Recently, Wang et al. [35] proposed a procedure to improve the plan quality, generating further models using in each subsequent model the best of the plans between those obtained from RP, and the original (or previous step) ones. They evaluated only the plan quality, without analysing the model summary results, showing an improved plan quality for the subsequent models.

Another comparison between RP models based on different initial plans has been recently published. In their work, Lin et al. [36] compared a model generated from clinical trial-and-error based plans on prostate, with a model generated for the same patients, whose plans were optimized according to a constrained hierarchical optimization procedure, a very complex and time consuming process able to produce Pareto optimal plans. They concluded that the RP model populated with this second group of plans improved significantly the model quality, in terms of R^2^ and DVH estimation bound width; the final clinical plan quality, however, was not significantly improved using the RP generated by this second group of plans, proving that the model quality does not necessarily translate into clinical plan quality. Those results are consistent with what presented in the current work.

Interesting is also the work of Fusella et al. [37], who evaluated the plan quality of the cohort used to train the model with plan quality metrics, in order to select the plans with the highest dosimetric quality, reducing in part the plan variability of the data feeding the model.

The main limitation of the current study refers to the restrictions of the RP implementation in the version 13.6 of Eclipse. In particular, during the RP based optimization, the generated mean dose value (for a mean objective) is not computed for the entire structure, but (presumably) for the part of the structure not overlapping the target. This generates mean dose values for overlapping OARs much lower than what achievable by the whole organ, increasing the sparing efforts in that structure as a function of the overlapping amount. This creates some inconsistency in the mean optimization objectives in the model, that should have been solved in a subsequent RP version. In the current work, the mean dose objectives were widely used.

## Conclusions

In summary, an RP model based on plans generated by a previous RP model improves the model quality showing better mathematical parameter results, and possibly the plan quality for clinical application. Moreover, each RP model is able to produce plans reflecting the input planning quality criteria: the RP input data should hence refer to the best plans according to a defined strategy, in order to obtain plans compliant with the institutional specific goals.

## Data Availability

Data supporting the findings of this work are available within the article.

## Appendix

### RapidPlan

The RP KBP engine consists of three main subsystems: *i)* a model training environment, *ii)* a dose volume histogram prediction environment, and *iii)* the generation of personalized dose-volume constraints for the plan optimization. The first is devoted to the data modelling of the plan and patient datasets and the training of the predictive model for each OAR. The second component aims to estimate the dose distribution as dose volume histograms, DVH, achievable for a certain new patient, based on the application of the predictive model. The third element derives from the estimated DVH and from a set of customizable rules associated with the model the actual dose-volume constraints to use for the dose plan optimization. These constraints could be of various nature: lower, upper, upper gEUD, mean dose, line objectives. This last constraint is an entire DVH, which is used as a single objective: in particular, the line objective is generally used from estimated DVH.

In the first phase, the RP engine performs the extraction of the dosimetric and geometric/anatomical data from a cohort of selected plans on different patients, and computes from those data a number of dosimetric and geometric metrics for each OAR. OAR specific dosimetric characteristics derive from the cumulative DVH, while the geometric information derive from a cumulative volume histogram of the Geometry-based Expected Dose (GED) metric, representing, in a simplified way, how far the various voxels in the structure are from the target surface, where the distance value is the amount of dose in a voxel, under a defined field geometry, given by the dose delivered to the targets. On DVH and GED, a principal component analysis is performed to determine the dosimetric and geometric features (principal components) which at best summarise the main characteristics of the input data. Some anatomical data are also extracted: the volume of the OAR, the fraction of the OAR volume overlapping with the targets, the fraction of OAR volume out of all the field projections, the volume of the joint targets. All those metrics are the input for the regression model, which, combining a dosimetric and a geometric feature (or a combination of them) resulted in a coefficient that can be used to estimate the OAR DVH for any new patient. A more detailed description of the RP and DVH estimation algorithm can be found in [26]. The version of the RP module used in this study was the 13.6.

After the model configuration, RP allows the user to analyse the model quality. On one side, the model training is evaluated through parameters specific for each structure and each plan in the model configuration. This evaluation phase is here considered as part of the configuration process, where possible outlier plans are identified and managed. On the other side, the model quality is given by a summary of goodness-of-fit and goodness-of-estimation statistics. These include, among others, per each trained organ at risk, the coefficient of determination R^2^ (goodness-of-fit), and the mean squared error MSE between original and estimate (goodness-of-estimation). The R^2^ describes how well the regression model represents the training plan data, by quantifying the variability in the data expressed by the model; its value ranges from 0 to 1: larger R^2^ indicates a better model fit, however, R^2^ values too close to 1 could be symptom of overfitting. The MSE describes how well the model is able to estimate the original DVH in a training plan, by measuring the distance between the original DVH and the mean of the upper and lower bounds of the estimated DVH; the closer the value is to 0, the better is the estimation capability of the model. The goodness-of-estimation statistics is generated during an internal cross-validation process of the model. The whole training set is firstly divided into 10 parts, and 10 models, one for each part, are trained. For each of those internal training sets, a different tenth of the whole plan set is left out and used for internal validation. The average of the validation statistics of these 10 internal models are finally summarized in the goodness-of-estimation statistics.
